# Prevalence of Bovine Fasciolosis and Its Economic Loss due to Liver Condemnation at Wolaita Sodo Municipal Abattair, Ethiopia

**DOI:** 10.1155/2019/9572373

**Published:** 2019-12-01

**Authors:** Adane Zewde, Yehualashet Bayu, Anteneh Wondimu

**Affiliations:** College of Veterinary Medicine, Haramaya University, PO Box 138, Dire Dawa, Ethiopia

## Abstract

Fasciolosis is a serious animal health problem in Ethiopia where cattle raising is very important to the local economy. A cross sectional study was carried out from November 2018 to February 2019 to estimate the prevalence of bovine fasciolosis and its associated risk factors as well as financial losses due to liver condemnation. A total of 247 cattle consisting of 219 males and 28 females were randomly selected and fecal sample collection for Fasciola* e*gg detection and postmortem liver inspection for adult liver flukes, were done. From the total of 247 cattle examined, the overall prevalence of bovine fasciolosis in the study area was 16.6% and 20.24% by coprological and postmortem examinations, respectively. The livers and bile ducts were examined for the adult flukes and the result showed that *F. hepatica *was frequently detected* Fasciola* sp. (72%) than *F. gigantica *(28%). In the study area, the prevalence of bovine fasciolosis between sex was significantly different (*p* < 0.05) with higher prevalence was recorded in female (57.1%) than male (15.5%). There was also significant association (*p* < 0.05) among different age groups for the prevalence of bovine fasciolosis with the highest prevalence in older (40.1%) than younger (18.8%) age groups. But, regarding origin and body condition the prevalence of bovine fasciolosis was not significantly associated (*p* > 0.05). The annual direct financial losses incurred due to fasciolosis were estimated around 1,505, 856 ETB ($43, 024.458). In conclusion, bovine fasciolosis is prevalent and economically important disease in the study area. Therefore, implementation of control and prevention strategy like, grazing managements, reducing the population of the intermediate host, diagnosis and treating sick animals using anthelmintic, is mandatory.

## 1. Introduction

Fasciolosis is a major problem that affects the productivity of livestock throughout the world [1, 2]. It is caused by the genus *Fasciola *which is commonly referred to as liver flukes [3]. The two species most commonly concerned as the etiological agents of fasciolosis are *Fasciola hepatica *(*F. hepatica*) and *Fasciola gigantica *(*F. gigantica*). Both species can infect a wide variety of domesticated animals, wild life and humans. *F. hepatica *found in temperate areas and in cooler areas of high altitude in the tropics and sub tropics, whereas *F. gigantica *which predominates in tropical areas [4]. Fasciolosis is commonly distributed in countries where cattle and sheep are raised and there is a niche for *Lymnaeid *snail and the disease repeatedly reported in different continent such as America, Australia, Europe, Asia and Africa [5].

The life cycle takes place in intermediate (IH) and definite hosts (DH). Definitive hosts include cattle, sheep, many other ruminants, equidae, swine and rabbits [6]. The spread of fasciolosis is largely dependent on the ecology of the snails which act as IH and serve as means of transmission to animals. *Lymnaea natalensis*, aquatic snails is important for *F. gigantica *in Africa, where as *Lymnaea natalensis truncatula*, is an amphibian, wide distribution worldwide, and the most common IH for *F. hepatica*. Adult flukes found in the bile duct, shed eggs into the bile then enter into the intestine to pass outside with feces [7]. The eggs hatch to motile, ciliated miracidium which infect the snail then, develop through the sporocyst and radial stage to the final stage, called cercaria. Cercaria shed from the snail and attaches to grass blades to form the infective metacercaria, which then ingested by the final host, excyst in the small intestine, migrate through the gut wall, cross the peritoneum and penetrate the liver capsule [6].

Fasciolosis is associated with liver damage and hemorrhage due to migration of flukes through the liver parenchyma. There is also haematophagic activity of the adult flukes and damage to the bile duct mucosa by their cuticular spines due to fluke residence in the bile duct [6, 7]. Diagnosis of fasciolosis may be established based on the epidemiology of the disease, observations of clinical signs, and information on grazing history [8]. However, confirmatory diagnosis is based on coproscopic examination in the laboratory and post-mortem examination of infected animals by the detection of flukes in the liver [9]. The treatment of fasciolosis, should be focused on the juveniles and adult fluke. In general Triclabendazole is effective against all developing stages over one week old. Moreover reduction of snail population is important measure in the control and prevention strategies [2].

In Ethiopia bovine fasciolosis is a widely distributed disease which imposes economic impact on livestock production particularly of cattle and sheep [10]. Study conducted by different scholars in Ethiopia indicated that the prevalence of bovine fasciolosis is ranging from 20.3% to 74.0% [11–14]. In the country, *F. hepatica *was shown to be the most important fluke species in livestock population with distribution over three quarter of the nation except in the arid northeast and east of the country. The distribution of *F. gigantica *was mainly localized in the western humid zone of the country that encompasses approximately one fourth of the nations [15, 16].

Fasciolosis also causes a substantial economic loss in cattle, goat, buffalo and sheep [17]. According to study reported by different researchers, the economic loss associated with affected liver condemnation due to fasciolosis in different area of Ethiopia is ranging from 86, 083.2 ETB ($2459.52) to 1,751,432 ETB ($50040.91) [12–14]. Since epidemiology of fasciolosis is dynamic and may change with years [18] and also the price of liver varies with years, it is therefore important to monitor its development to determine trends in prevalence and estimate financial loss associated with liver condemnation, due to fasciolosis. Though there fasciolosis, is economically important disease there is scarcity of well-documented information on the occurrence of fasciolosis and also financial loss associated with liver condemnation among cattle in Wolaita soddo, Ethiopia. Therefore the aim of this study was to determine the current prevalence of bovine fasciolosis and its associated risk factors as well as financial losses due to liver condemnation.

## 2. Materials and Method

### 2.1. Study Area

The study was conducted in Wolaita zone, at Wolaita Soddo town Municipal Abattoir, which is located in the Southern Nations Nationality and people's regional state, Southern Ethiopia. Wolaita Zone has a total of 4471.3 Km^2^ areas, and is located between 6.40–7.20 N and 37.4–38.20 E and 383 kms far from Addis Ababa and 165 kms far from Hawassa town. The Wolaita Soddo town lies between the altitude range of 2000–2500 meters above sea level and annual average rainfall ranges from 450 mm to 1446 mm. The mean annual maximum and minimum temperature are 26.6°C and 11.4°C, respectively. The livestock population of the area comprises about 128,919 cattle, 29,191 sheep, 4,606 goats, 4,124 equines and 55,278 poultry. The predominant farming system is mixed livestock and crop production system [19].

### 2.2. Study Population

The study population included all cattle brought for slaughter to Wolaita Soddo municipal abattoir during the study period. This population comprised of cattle of different sex, age, body condition and originating from Gofa, Humbo and Boreda.

### 2.3. Study Design

The cross sectional study was conducted from November, 2018 to February, 2019. Fecal sample collection and an active abattoir survey, were carried out for coprological examination and postmortem examination of liver to determine the prevalence and the economic impact of fasciolosis in the study area.

### 2.4. Sample Size Determination

The sample size of the study was determined using the formula given by Thrustfied [20]. The expected prevalence of 20.3% was considered from previous study reported by Mekonnen and Geta [12], confidence level of 95% and required absolute precision of 5% was used.

(1) $$\begin{array}{c} N=\frac{1.96^{2}*\;\text{pexp}\;\left(1-\;\text{pesp}\right)}{d^{2}}, \end{array}$$

where *N* = required sample size. pexp = expected prevalence = 20.3%, *d* = desired absolute precision = 5%. Accordingly, a total of 247 cattle were used for this study.

### 2.5. Sampling Techniques

The survey was conducted based on randomized systematic sampling method on the bases of entrance to lairage and code numbers written on their body. The slaughtering process was done 5 days per week. Based on the schedule, regular visit (two days per week) was made to conduct coprological and postmortem examination in Wolaita municipal Abattoir. Each day on an average of about 62 cattle slaughtered at Wolaita Manicipal abattoir. Averages of 8 cattle were sampled each day and a total of 247 heads of cattle was selected. During ante mortem inspection each cow was inspected by routine procedures and each was subjected to post mortem examination, for liver examination.

### 2.6. Study Methodology

Ante-mortem inspection: Data collected during ante-mortem examination included age, sex, body condition score and origin of animals. Animals were categorized into three age groups as young (<6 years), adult (6–8 years) and old >8 years, based on their dentition [21]. The data on body condition score was collected on scale of 1–9 (1-marked emaciation; 2-prominent transverse process and spines; 3-prominent dorsal spines, hips point, tail head and ribs; 4-ribs and hips clearly visible; 5-ribs visible little fat cover and spines barely visible; 6-animal smooth and well covered, dorsal spines cannot be seen; 7-animal smooth and well covered, but fat deposits are not marked, but round; 8-fat cover on critical areas, transverse process cannot be seen; 9-heavy deposits of fat clearly visible on tail head, brisket; dorsal spines, ribs hooks and pins fully covered and cannot be felt even with firm pressure) [22]. For the purpose of data analysis, cattle with body condition score of ≤4 were considered as poor, body condition score of 5–6, considered as medium and those with body condition scores above 6 were considered as good.

### 2.7. Coprological Examination

Before sampling; an identification number was given to each cattle that were randomly selected in the abattoir. Then fecal samples were collected directly from the rectum of each cattle, using disposable plastic gloves and placed in clean universal bottle and each sample was labelled with cattle identification number, age, sex, BCS, date and origin. Then the samples were preserved with 10% formalin solution. The samples were taken to Wolaita regional laboratory; then coproscopic examinations were performed to detect *Fasciola* eggs using standard sedimentation technique, as described by Hanson and Perry [23]. Morphological identification of eggs of* Fasciola* sp was conducted according to Urquhart et al. [7].

### 2.8. Post Mortem Examination

Animals, whose samples taken and examined during the ante mortem examination, were further supervised for their livers and bile duct. Careful examination by visualization and palpation of the entire organ, followed by incision along the bile ducts of the lobes, was done [24]. Liver parenchyma and major bile ducts were examined for the presence of immature and adult* Fasciola* parasites, respectively. Species are identified based on size and morphological characteristics according to Soulsby [25].

### 2.9. Economic Loss Assessment

Direct economic loss was resulted from condemnation of liver affected by fasciolosis. Generally, all infected livers with fasciolosis were considered to be unfit for human consumption. The annual losses from liver condemnation were assessed by considering the overall annually slaughtered animals in the abattoir. It was estimated from retrospective abattoir records. The average number of cattle slaughtered at the abattoir was 14,880 per year based on two consecutive year recorded data in the abattoir, while retail market price of an average size liver was 500 Birr ($14.29). It was determined from the information collected from butchers' in Wolaita Sodo town. The information obtained was subjected to mathematical computation as follows:

(2) $$\begin{array}{c} \text{ALc}\;=\;\text{CSw}*\text{LC}*\;P500*, \end{array}$$

where = ALc = annual loss from liver condemnation. CSw = mean annual cattle slaughtered at Wolaita municipal abattoir. LC = mean cost of one liver in Wolaita town. P = prevalence rate of the disease at the study abattoir. Accordingly ALc = 14,880 cattle ∗ 500 (birr)  ∗ 20.24% ALc = 14880 ∗ 500 ETB ∗ 0.2024. Alc = 1,505,856 ETB or $43024.45.

### 2.10. Data Analysis

Data exposed to quantitative analysis using descriptive statistics. The data were recorded on specially designed forms and preliminary analysis was done in Microsoft Excel (2007). The outcome variables were the cases of fasciolosis detected during routine post-mortem inspection and fecal examination of *Fasciola* sp. and eggs. STATA Version 11 was used for logistic analysis. The prevalence of fasciolosis was calculated as the number of cattle found infected with *Fasciola* sp. expressed as a percentage of the total number of previously selected cattle. The economic importance of the disease was assessed from the total number of livers condemned due to fasciolosis and the total number of animals slaughtered during the study period, calculated on an annual basis, using both present and retrospective data obtained from the slaughter house.

## 3. Result

Though this study used low sample size (247) as the limitation of the study, this was calculated by Thrustfied [20] standard formula using the expected prevalence of 20.3% reported by Mekonnen and Geta [12] in the same study area. Out of 247 indigenous cattle breeds that were slaughtered at Wolaita Sodo municipal abattoir, 41 animals were found infected with liver fluke with the prevalence of 16.6% based on coprological examination whereas 50 animals were found infected with liver fluke with the prevalence of 20.24% based on postmortem examination. In general postmortem finding has revealed better result than coprological examination (Table 1).

Detail postmortem examination of 50 animals infected with liver fluke, 36 (72%) livers were harbored *F. hepatica*, and 14 (28%) livers harbored *F. gigantica*. The distribution and prevalence of *Fasciola *species was different in different origins (districts) of animals. The highest prevalence of *F. hepatica *(72%) and *F. gigantic *(28%) was observed in Gofa and the lowest prevalence of *F. hepatica *(10.53%) and *F. gigantica *(14.29%) was observed in Humbo and Boreda respectively (Table 2).

Postmortem prevalence of bovine fasciolosis within different origin indicated that the highest prevalence (23.6%) was found in Gofa and the lowest prevalence (15.8%) was recorded in Humbo wereda. This difference was not statistically significant (*P* > 0.05). The current study indicated that the higher prevalence was recorded in female (57.1%) than male (15.5%), and revealed sex had significant effect (*P* < 0.05) on the prevalence of bovine fasciolosis. There was also a statistically significant difference (*P* < 0.05) in the prevalence of bovine fasciolosis in different age groups. The highest prevalence (40.1%) was found in old animals and the lowest prevalence (8.0%) was found in young animals. Though the highest prevalence (35.2%) was found in animals with poor body condition scores and the low prevalence (7.7%) in moderate body conditioned animals, there was no significant difference in the prevalence of bovine fasciolosis within different body condition scores (Table 3).

Higher coprological prevalence was recorded in cattle purchased from Gofa (18.2%) and Boreda (18.0%) followed by Humbo (13.2%). This different prevalence of bovine fasciolosis among origin was not statistically significant (*P* > 0.05). The current study indicated that the lower prevalence was recorded in male (13.2%) than female (42.9%), and this difference was statistically significant (*P* < 0.05) on the prevalence of bovine fasciolosis. The highest prevalence (33.3%) was found in old animals followed by adult (15.6%) and young animals (4.0%). This difference was also statistically significant (*P* < 0.05) for the prevalence of bovine fasciolosis in different age groups. There was no statistical significant association for the occurrence of bovine fasciolosis among different body condition scores, but the highest prevalence (29.7%) was found in animals with poor body condition scores followed by good (15.2%) and moderate (7.7%) body conditioned animals (Table 4).

The economic significance of fasciolosis was analyzed based on the information obtained during post mortem examination and interview. The 50 infected livers of cattle imposed an estimated total loss of 25,000 ETB ($714.3). In the study abattoir the average annual cattle slaughtered rate was estimated to be 14,880 while mean retail price of bovine liver in Wolaita Soddo town was 500 ETB ($14.29). A total of 1,505,856 ETB ($43,024.45) annual losses were calculated from organ condemnation using the current abattoir prevalence (20.24%).

## 4. Discussion

The obtained results in the study indicate fasciolosis is an economically important disease in the study area. In current study higher number for fasciolosis were detected by postmortem examination compared with coprological examination; this is similar with the finding in Wolaita [12]. But, the coprological result was lower than 42.25–72.8% findings from different parts of Ethiopia [14, 26, 27]. This difference could be explained by the fact that ecological difference affected the rate of prevalence of the fasciolosis, significantly. Postmortem examination's result (20.24%) in the present study was lower than that of the prevalence in Sheno (74.0%) and in Sinnana (47%) [14, 28, 29]. This could be attributed to ecology, climate and management system differences [30]. The higher postmortem examination result was consistent with the report from Sinnana [28]. This might be due to the need of longer prepatent period from 8–15 weeks after infection for the egg to appear in the feces [2].

Postmortem examination of the infected livers revealed that *F. hepatica *(72%) was the most prevalent species compared to *F. gigantica *(28%). The current finding was consistent with different research in different parts of Ethiopia [13, 29, 31–34]. This was associated with the existence of favorable ecological condition for *L. truncatula *which is IH host of *F. hepatica *in the study area. However, Fufa et al. [35] stated that *F. gigantica *was the most common liver fluke species affecting cattle at the same study area. The infection of cattle with *F. gigantica *in the present study could be due to the reason that the cattle slaughtered in the abattoir were originated from lowlands and middle altitude zone flood prone areas, drainage ditches which are favorable habitat to *L. natalensis *[7].

In the present study, the prevalence of fasciolosis in cattle was affected by sex of animals. However, the work done by different scholars concluded that sex has no impact on the infection rate and hence both male and female are equally susceptible and exposed to the disease [36–38]. High infection rate in females can be multifactorial like high stress during parturition period, weak and malnourished making them more susceptible to infection or due to the feeding conditions i.e. females are generally being let loose to graze freely in pastures [39]. The other possible reason could be that the most of people traditionally feed their lactating cows with grasses during dry season which are grown around rivers and marshy areas in order to improve milk yield as suggested by Tilahun et al. [40]. However, some study revealed that male cattle are more prone to fasciolosis than female [41].

The results of the present study revealed that age has a significant effect on the prevalence of bovine fasciolosis. Different works reported similar finding with the present work [35, 36, 42]. In the current study higher prevalence was recorded in older cattle than younger. This may be due to the longer exposure of older cattle when outside for grazing and due to younger cattle being kept indoor and geting good management. And also as the cattle get older, their immunity may be decreased [2].

The prevalence of fasciolosis was higher in poor body conditioned animals than that of medium and good body conditioned animals. Similar finding was also reported by Bekele et al. [43] and Belay et al. [44]. The reason behind this may be due to a lack of regular deworming of animals; poor body condition in cattle thatis manifested in chronic stage of fasciolosis; Inadequate nutrition and concurrent infection of the animals with other bovine pathogens that could enhance the effects of the flukes for the emaciation of the animals. Both postmortem examination and coprological prevalence showed the prevalence of fasciolosis, which was not significantly different in the three districts except, of slightly higher prevalence in case of cattle from Gofa. This could be due to similarity in agro-ecology and management system.

Fasciolosis causes economic losses in livestock as the result of mortalities, abortions, retarded growth, reduced meat and milk production, condemnation of infected livers and emaciated carcass [45]. A total sum of money 1,505,856 ETB ($43,024.45) was lost due to liver condemnation associated with fasciolosis in the present study. This result showed that fasciolosis causes significant losses in the study area, at large. These findings were by far higher than 154,188 ETB ($4,405.37), 215,000 ETB ($6,142.85), 267,512 ETB ($7643.2) and 154,490ETB ($4414) per annum in cattle due to fasciolosis reported by different scholars in Ethiopia and some were in Africa [29, 47–49], but lower than 3,003,488.14 ETB ($85,813.94) reported by Terefe et al. [49] in Jimma municipal abattoir. This is probably due to the ecological and climatic difference between localities, local liver cost, and yearly inflation may be also considered.

## 5. Conclusion

The current finding showed that bovine fasciolosis is the most common and economically important parasitic disease affecting the health and productivity of animals in the study area. It causes great economic losses as a result of condemnation of infected livers. The most prevalent *Fasciola *sp. obtained in condemned livers was *F. hepatica*. Sex and age are among associated factors on prevalence of bovine fasciolosis. In the study, around 24,700 Ethiopian birr (ETB) ($705.72) was lost due to *Fasciola* disease. A total of 1,505,856 ETB ($43,024.45) annual losses were also calculated from liver condemnation. This indicates, fasciolosis is a serious economically important disease to the livestock industry that warrants due attention in the study area. Therefore:- strategic prevention of bovine fasciolosis should be initiated by regular deworming of animals before and after rainy season and practicing effective drainage system in order to control the snail.

## Figures and Tables

**Table 1:** Comparison of the prevalence of bovine fasciolosis based on coprological and post mortem examination.

Results	Coprological examination (%)	Post mortem examination (%)
Positive	41 (16.6)	50 (20.24)

**Table 2:** Prevalence of *Fasciola *sp. based on origin of the animal.

*Fasciola* species	Number of detected	Origin					
		Gofa	Humbo	Boreda	Crude OR	*P*-value	95% CI
*F. hepatica *(*%*)	36 (72%)	18 (50.00)	8 (10.53)	10 (16.39)	0.85	0.425%	(0.58–1.26)
*F. gigantica *(%)	14 (28%)	8 (57.14)	4 (28.57)	2 (14.29)			
Total	50 (20.24%)	26	12	12			

**Table 3:** Postmortem prevalence of bovine fasciolosis based on their origin, sex, age and body condition.

Category	Variable	Number of animal examined	Number of positive	Prevalence	Crude OR	*P*-value	95% CI
Origin	Gofa	110	26	23.6	0.85	0.43	(0.6–1.3)
	Humbo	76	12	15.8			
	Boreda	61	12	19.7			
Sex	Male	219	34	15.5	7.25	0.001^∗^	(3.2–6.7)
	Female	28	16	57.1			
Age	<6 years	25	2	8.0	2.83	0.003^*^	(1.4–1.6)
	6–8 years	192	36	18.8			
	>8 years	30	12	40.1			
Body condition	Poor	37	13	35.2	0.72	0.098	(0.5–1.1)
	Medium	26	2	7.7			
	Good	184	35	19.0			

CI-confidence interval ^∗^significant.

**Table 4:** Coprological prevalence of bovine fasciolosis based on their origin, sex, age and body condition score.

Category	Variable	Number of animal examined	Number of Positive	Prevalence	Crude OR	*P*- value	95% CI
Origin	Gofa	110	20	18.2	0.9	0.9	(0.6–1.5)
	Humbo	76	10	13.2			
	Boreda	61	11	18.0			
Sex	Male	219	29	13.2	4.9	0.001^*^	(2.1–11.4)
	Female	28	12	42.9			
Age	<6 years	25	1	4.0	2.9	0.003^*^	(1.4–6.2)
	6–8 years	192	30	15.6			
	>8 years	30	10	33.3			
Body condition	Poor	37	11	29.7	0.7	0.089	(0.5–1.1)
	Medium	26	2	7.7			
	Good	184	28	15.2			

CI-confidence interval ^∗^significant.

## Data Availability

The raw data used to support the findings of this study are included within the supplementary information file(s).

## Abbreviations

ALC: Annual loss of liver condemnation
ETB: Ethiopian Birr
FAO: Food and agricultural organization
IH: Intermediate host
MASL: Meter above sea level.

## Additional Points

I, the undersigned, declare that this research is original work, has not been submitted for publication anywhere and all sources of materials used for this research have been duly acknowledged.

## Supplementary Materials

Supplementary Materials Supplementary file contains research raw data coded in excel for analysis of the research findings. The raw data includes Ante-mortem and postmortem inspection results: Ante-mortem examination contains different variables such as age, sex, body condition score and origin of animals; and coprological examination. Animals were categorized into three age groups as young (<6 years), adult (6-8 years) and old >8 years. Cattle with body condition score of < 4 were considered as poor, body condition score of 5 to 6 considered as medium and those with body condition scores above 6 were considered as good. These all were variables to determine the risk factors for the prevalence of bovine fasciolosis. Coprological examinations were also performed to detect Fasciola eggs. Animal with fasciola egg considered as positive, animal without fasciola egg considered as Negative. At post mortem examination Liver parenchyma and bile ducts were examined for the presence of immature and adult Fasciola parasites respectively; animal with the presence of fascila parasite considered as positive result and wirhout fasciola parasite considered as negative result. Species were also identified as *F. hepatica* and *F. gigantica*.
